# A139 EARLY VIDEO CAPSULE ENDOSCOPY VERSUS COLONOSCOPY FOLLOWING NEGATIVE ESOPHAGOGASTRODUDENOSCOPY IN PATIENTS WITH SUSPECTED UPPER GASTROINTESTINAL BLEEDING: PRELIMINARY DATA FROM A RANDOMIZED CONTROLLED TRIAL

**DOI:** 10.1093/jcag/gwad061.139

**Published:** 2024-02-14

**Authors:** K Patel, D Yang, T Krahn, S Wesilenko, B Halloran, S Zepeda-Gomez

**Affiliations:** University of Alberta, Edmonton, AB, Canada; University of Alberta, Edmonton, AB, Canada; University of Alberta, Edmonton, AB, Canada; University of Alberta, Edmonton, AB, Canada; University of Alberta, Edmonton, AB, Canada; University of Alberta, Edmonton, AB, Canada

## Abstract

**Background:**

Esophagogastroduodenoscopy (EGD) is recommended for initial endoscopic evaluation of patients with suspected upper gastrointestinal bleeding (UGIB). When this is negative, the standard recommendation is to perform a colonoscopy, despite low diagnostic yield of less than 5%. In these patients, the question remains as to whether small bowel investigations would be of higher yield prior to colonoscopy.

**Aims:**

To compare the diagnostic yield between early video capsule endoscopy (VCE) versus colonoscopy after a negative EGD in patients with suspected UGIB.

**Methods:**

This is preliminary data from a prospective randomized control trial (RCT) of adult patients with suspected UGIB (melena and hemoglobin drop of more than 20 g/L within 48 hours of admission). Patients at a single centre were enrolled prior to initial EGD; those with negative EGD were included and randomized to either colonoscopy (next day) or early VCE (immediately after EGD). Patients with a confirmed source of bleeding in their group required no further testing, but those without underwent further testing with the alternative (VCE or colonoscopy). We evaluated patient outcomes, including bleeding localization time, hospitalization duration, procedure count, complications and rebleeding rates.

**Results:**

A total of 19 adult patients have been enrolled, of which 12 had negative EGD and were randomized to either VCE or colonoscopy, with six patients in each arm (Figure 1). In the VCE group, 100% (6/6) of patients had bleeding sources detected by early VCE, with 50% (3/6) of them undergoing endoscopic treatment. In the colonoscopy group, only one patient had a positive finding (1/6), the rest underwent subsequent VCE. The VCE identified the bleeding source in 80% (4/5) of patients with negative colonoscopy. Patient are outlined in Table 1.

**Conclusions:**

Based on our preliminary data, the diagnostic yield of early and subsequent VCE was higher when compared to colonoscopy after initial negative EGD in patients with suspected UGIB.

Table 1. Summary of findings and patient outcomes

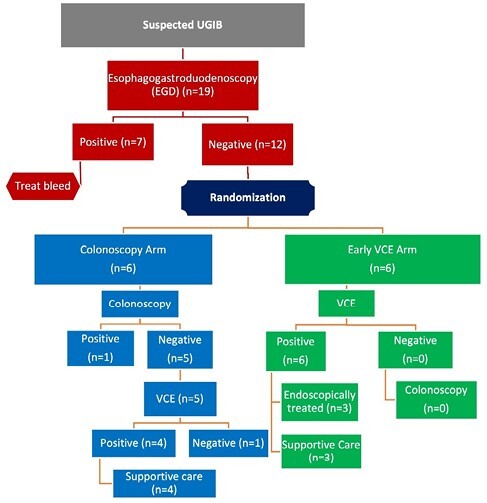

**Funding Agencies:**

Medtronic Canada (Brampton, ON, CA) will provide the video capsule in the treatment group. This is the only equipment funding required in this study.

**Table:** 

OUTCOMES	Early VCE Arm	Colonoscopy Arm	All participants
Bleeding localized after first test, % (n)	100 (6/6)	17 (1/6)	58 (7/12)
Average time to localization of bleed (days) ± standard deviation	5.33 ±2.46	6 ±2.31	5.67 ±1.62
Average length of hospital stay (days) ± standard deviation	5.33 ±0.97	5.17 ±1.55	5.25 ±0.874
Average number of procedures	2.67	3.17	2.92
Etiology of bleeding	-Actively bleeding AVMs in small bowel – 33% (2/6) -Non-bleeding AVMs – 50% (3/6) -Active bleeding in right colon – 17% (1/6)	-Clean based longitudinal ulcer in transverse colon – 17% (1/6)	

